# Linking community-acquired images to national data in the absence of interoperable systems and unique identifiers

**DOI:** 10.23889/ijpds.v9i5.2726

**Published:** 2024-09-18

**Authors:** Claire Tochel, Heather Anderson, Miguel Bernabeu, Baljean Dhillon, Malihe Javidi, Tom MacGillivray, Alice McTrusty, Jon Penny, Niall Strang, Andrew Tatham, Jordan Watson, Sam Watts

**Affiliations:** 1 University of Edinburgh; 2 Princess Alexandra Eye Pavilion; 3 Royal College of Surgeons Edinburgh; 4 Glasgow Caledonian University

## Objective & Approach

Routine optometry examinations, which may include retinal images, have been free to everyone in Scotland since 2006. Images and patient data are stored locally on non-interoperable systems, usually without unique identifiers. The Scottish Collaborative Optometry-Ophthalmology Network for e-research (SCONe) aimed to create a secure, longitudinal repository collating community-acquired images linked to national, routinely-collected healthcare data. We developed bulk export processes to retrieve images and patient data from current and archived systems and a series of deterministic and probabilistic algorithms to match images to patients.

## Results

We successfully exported images and patient data from several commonly used systems. Exact matching of available data between image and clinical systems within practices linked 78% of images (IQR 71.2-84.1). Our algorithms increased match rate to 90% (IQR 83.5-98.6) of images, efficiently reducing risk of systemic bias in the cohort. Match method is included in the repository metadata to facilitate quality assurance checks after national linkage and pseudonymisation. To date over 600k images have been processed and delivered to the National Safe Haven for linkage to healthcare data.

## Conclusions & Implications

Breakthrough research into early detection and pre-symptomatic disease can be facilitated by harnessing community-acquired retinal images which capture normal ageing and disease progression. Rather than await interoperability and widespread use of unique identifiers, this has been achieved through innovative solutions to real-world issues around time-pressured clinical data entry and non-standardised data storage. The SCONe repository continues to grow. It is currently supporting ocular disease prediction research and offers the potential for much more.

